# Accelerating root system phenotyping of seedlings through a computer-assisted processing pipeline

**DOI:** 10.1186/s13007-017-0207-1

**Published:** 2017-07-13

**Authors:** Lionel X. Dupuy, Gladys Wright, Jacqueline A. Thompson, Anna Taylor, Sebastien Dekeyser, Christopher P. White, William T. B. Thomas, Mark Nightingale, John P. Hammond, Neil S. Graham, Catherine L. Thomas, Martin R. Broadley, Philip J. White

**Affiliations:** 1 Ecological Sciences, The James Hutton Institute, Invergowrie, Dundee, DD2 5DA UK; 2 Cell and Molecular Sciences, The James Hutton Institute, Invergowrie, Dundee, DD2 5DA UK; 3 Elsoms Seeds Ltd, Pinchbeck Rd, Spalding, PE11 1QG UK; 4 School of Agriculture, Policy and Development, University of Reading, Whiteknights, PO Box 237, Reading, RG6 6AR UK; 5 Plant and Crop Sciences, School of Biosciences, University of Nottingham, Sutton Bonington Campus, Loughborough, LE12 5RD UK; 6 Distinguished Scientist Fellowship Program, King Saud University, Riyadh, 11451 Kingdom of Saudi Arabia

**Keywords:** Root, Phenotyping, Error, Pipeline, Barley, Brassica

## Abstract

**Background:**

There are numerous systems and techniques to measure the growth of plant roots. However, phenotyping large numbers of plant roots for breeding and genetic analyses remains challenging. One major difficulty is to achieve high throughput and resolution at a reasonable cost per plant sample. Here we describe a cost-effective root phenotyping pipeline, on which we perform time and accuracy benchmarking to identify bottlenecks in such pipelines and strategies for their acceleration.

**Results:**

Our root phenotyping pipeline was assembled with custom software and low cost material and equipment. Results show that sample preparation and handling of samples during screening are the most time consuming task in root phenotyping. Algorithms can be used to speed up the extraction of root traits from image data, but when applied to large numbers of images, there is a trade-off between time of processing the data and errors contained in the database.

**Conclusions:**

Scaling-up root phenotyping to large numbers of genotypes will require not only automation of sample preparation and sample handling, but also efficient algorithms for error detection for more reliable replacement of manual interventions.

## Background

There will be a demand for more food as the human population increases [1–3]. However, many of the resources used for food production, such as irrigation water and mineral fertilisers, are becoming relatively more expensive [4]. Additional costs could restrict agricultural inputs and limit crop production [5]. One strategy to mitigate this is to breed resource-efficient crops that have similar, or greater, yields with less resource input [2, 3]. Since the acquisition of water and mineral elements is a function of the root system, it is anticipated that a “second green revolution” to develop resource-efficient crops would focus on the improvement of root systems for resource capture [4, 6–10]. Developing resource-efficient crops requires rapid phenotyping of root system architectures of many genotypes in a cost-effective manner [4].

There are many techniques for quantifying aspects of root system architecture that can be applied in the field or in controlled environments [4, 11–14]. Most modern approaches rely on imaging root systems, whether using digital cameras, flatbed scanners, X-rays or magnetic resonance, and use computer software to analyse the images collected [15–21]. Adoption of some of these techniques by low-cost, high-throughput phenotyping (HTP) programmes is restricted by lack of automation and, therefore, staff costs (e.g. field phenotyping; analysis of soil cores), the cost of infrastructure or the availability of specific expertise (e.g. X-ray tomography or Magnetic Resonance Imaging), the requirement for bulky equipment (e.g. 3D scanning systems) or the necessity to phenotype mature plants [14, 22, 23]. By contrast, simple pouch-and-wick or petri-dish systems appear to be suitable for screening root architectures of large numbers of seedlings in a rapid and cost-effective manner, provided plant growth is separated from imaging the root system [14, 24–29]. Such systems allow the lengths, branching and angles of different root types to be estimated [14, 27], which form the basis of models to generate root system architectures of crop genotypes [30–32] and their consequences for resource capture [33, 34]. Even the measurement of simple root traits appears to have predictive value for breeding crops with greater yields and stress tolerance [5, 18, 35, 36]. For example, (1) Saengwilai et al. [37] observed that maize (*Zea mays* L.) genotypes with fewer crown roots exploited a deeper soil volume, acquired more nitrogen (N) and yielded more than other genotypes on soils with low N availability, (2) Lynch and Brown [38] showed topsoil foraging increases phosphate uptake efficiency in common bean and (3) Thomas et al. [14] observed that primary root length of oilseed rape (oilseed rape; *Brassica napus* L.) seedlings in a HTP pouch-and-wick system correlated with crop emergence in three out of five field trials and with seed yield in four out of six field trials.

In this paper we describe (1) a low-cost HTP system for phenotyping seedling root architecture, (2) a semi-automated image capture and curation pipeline, (3) a new algorithm for correcting manual placement of markers on seeds and root tips and an optimal path algorithm similar to that used by other tracing software [41] for tracing roots without prior knowledge of relationships between seeds and root tips that allow data on root number per seed, root length per seed and root angles to be estimated rapidly for large populations with minimal user input. Estimates of equipment costs and processing time per seedling are provided, from which costings for infrastructure and running costs might be produced. The types and frequency of errors produced by image analysis using the software developed for this pipeline, ArchiPhen, are described and the effects of errors are assessed in comparisons with similar software.

## Methods

### Growth system

**Fig. 1:**
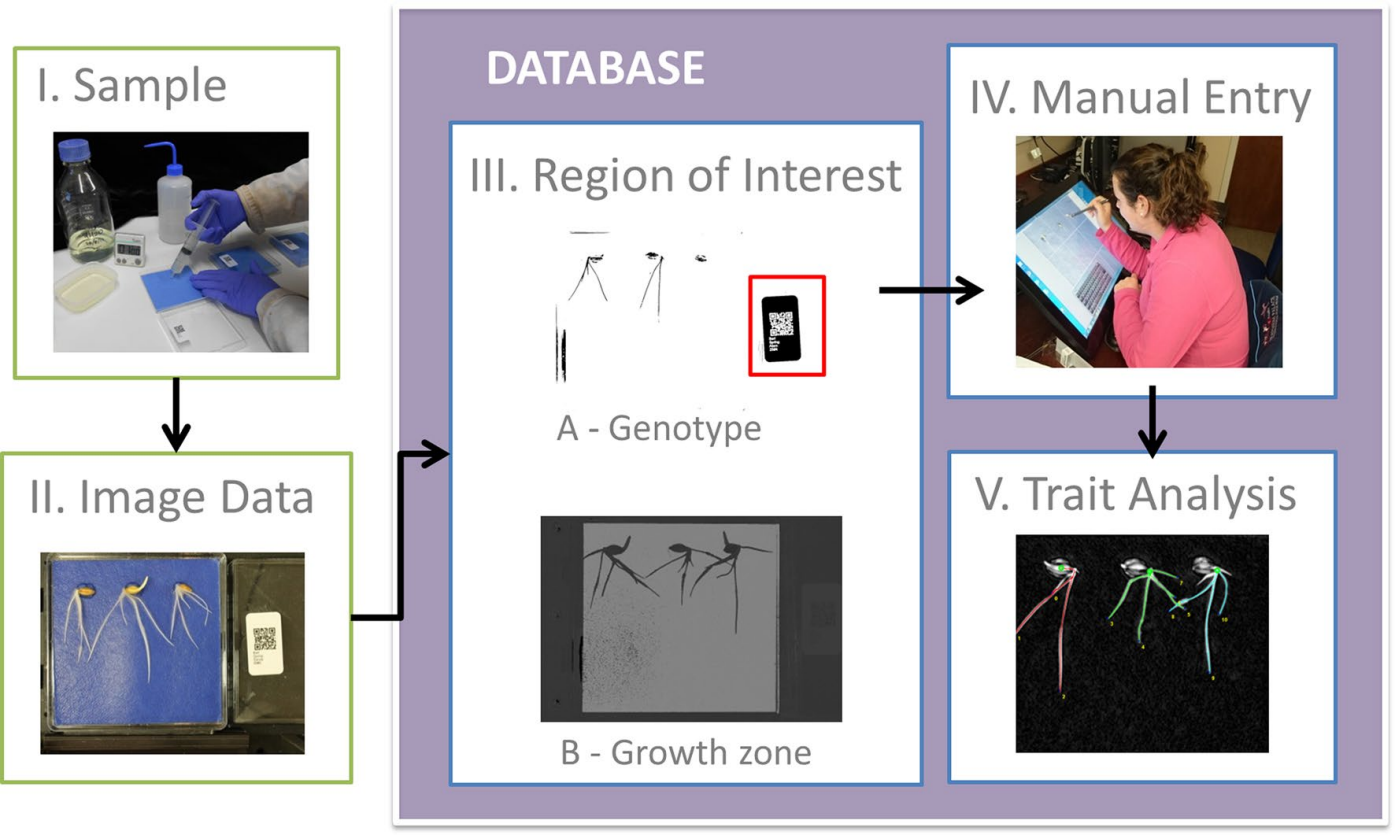
Root phenotyping pipeline consisting of five steps. (*I*) Plants are grown on germination paper placed into a petri dish; (*II*) opened petri dishes containing seedlings are imaged with a camera placed vertically at a height of 39 cm above the petri dish to include all the germination paper as well as the part of the lid of the petri dish containing the QR code; (*III*) image processing algorithms are used to identify the QR code and the region of the blue germination paper containing roots; (*IV*) the location of seeds and root tips are marked on cropped images using a manual stylet and customised software; (*V*) a suite of algorithms are employed to trace roots and extract root traits from the tracings

**Fig. 2:**
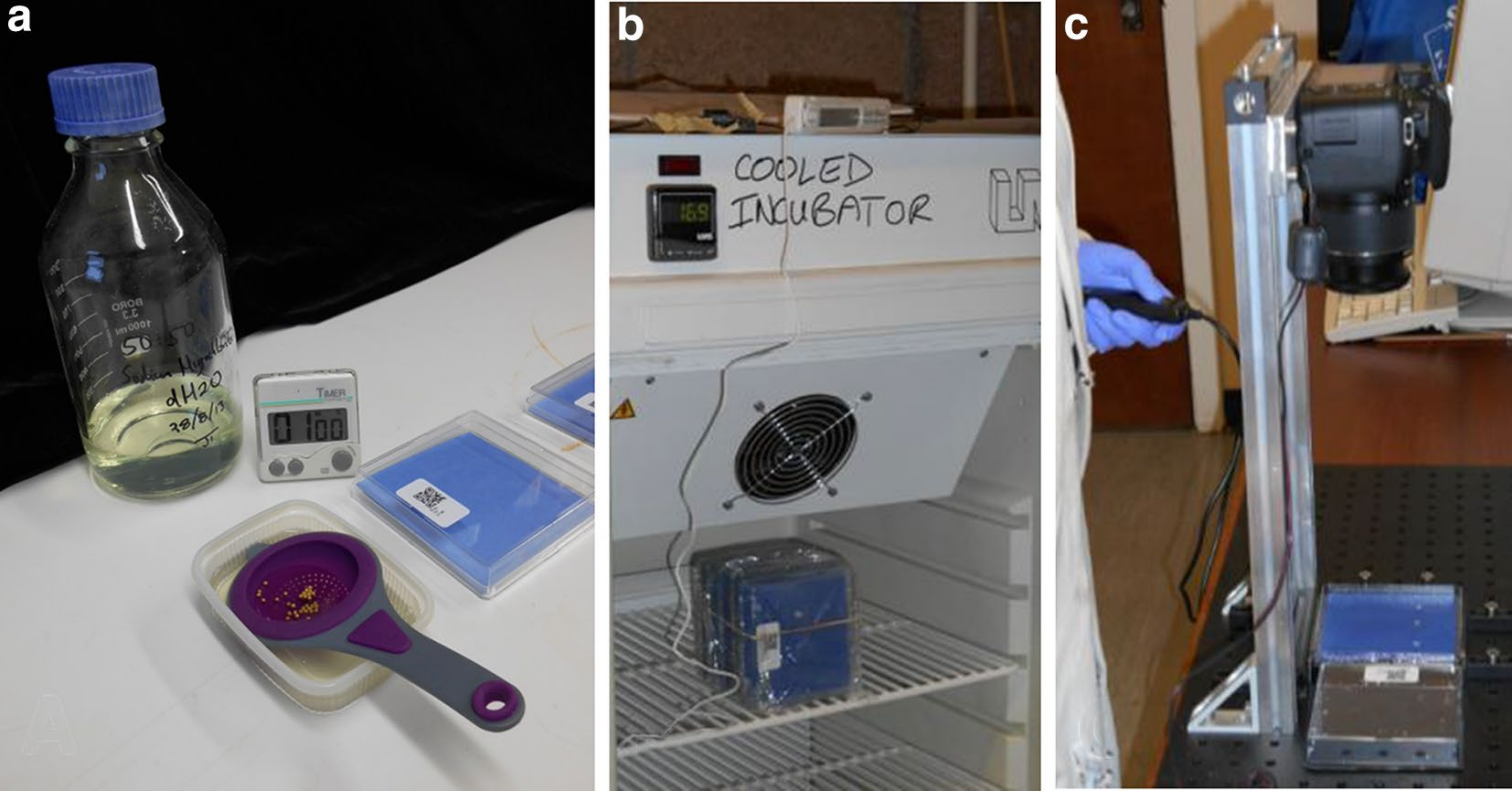
Low cost root phenotyping is achieved by **a** using inexpensive consumables such as germination paper, petri dishes and limited lab reagents; **b** growing seedling in a laboratory incubator; and **c** using an inexpensive DSLR camera and custom made software for handling the data

Plants were grown on germination paper placed in a petri dish (Fig. 1). Seedling root systems of an oilseed rape (oilseed rape; *Brassica napus* L.) genetic mapping population (TNDH; n = 204 genotypes; [39]), generated through anther culture of the F_1_ generation of a cross between Tapidor (a European winter oilseed rape cultivar) and Ningyou 7 (a Chinese semi-winter oilseed rape cultivar), and a barley (*Hordeum vulgare* L.) association genetic mapping population (AGOUEB; n = 304 Spring barley genotypes and n = 282 Winter barley genotypes; [40]) were studied. Seeds were surface sterilised for 1 min in 50% v/v sodium hypochlorite. Three surface-sterilised seeds of a specific genotype were attached to blue germination paper (dimensions 99 mm × 99 mm; SD7640, Anchor Paper Company, St Paul, MN, USA) using a drop of wallpaper paste (Solvite, Henkel Limited, Winsford Cheshire, UK). The germination paper with seeds attached was then placed in a square petri dish (length × width × depth = 100 mm × 100 mm  × 18 mm; Camlab, Cambridge, UK) and 10 mL distilled water was added (Fig. 2a). The lid of each petri dish was labelled with a QR code containing details of the plant species, the plant population, the genotype, and the replicate number of the petri dish (Fig. 2). The experiment consisted of five petri dishes of each genotype (n = 15 plant replicates). Dishes were wrapped in clingfilm to maintain humidity and placed upright in an unlit controlled environment cabinet (LMS Cooled Incubator Series 1a Model 201; LMS Ltd, Sevenoaks, Kent, UK) set at 15 °C. Petri dishes containing seedlings were removed from the cabinet, seedlings photographed and paper rewetted with 2 mL water 5 and 8 days after sowing (DAS) for oilseed rape or 4 and 5 DAS for barley. Screening of the TNDH oilseed rape population was performed sequentially in five batches containing all replicates of 20, 26, 44, 62 and 48 genotypes, respectively. Screening of the Spring barley genotypes of the AGOUEB population was performed sequentially in eight batches containing all replicates of 12, 16, 57, 50, 69, 56 and 44 genotypes, respectively. Screening of the Winter barley genotypes of the AGOUEB population was performed sequentially in five batches containing all replicates of 56, 56, 56, 62 and 52 genotypes, respectively.

### Image acquisition

Opened petri dishes containing seedlings were placed on a stage custom built to accommodate both the bottom, containing seedlings, and the lid, with the QR code, of the petri dish (Fig. 2c). The stage was assembled on a 60 × 60 cm solid aluminium breadboard (Thorlabs, MB6060/M) with optical rails (Thorlabs, XE25 metric series) for rapid placement of the petri dish beneath the camera (Canon 550D EOS). The camera was placed vertically at a height of 39 cm above the petri dish and was fitted with a Canon EF-S 18–55 mm f/3.5–5.6 IS STM Lens. The height and objective magnification was adjusted for the field of view to include all the germination paper as well as the part of the lid of the petri dish containing the QR code. The bottom of the perti dish was elevated by 2 cm so that the seedling root system and the QR code were both at the same height. The focus of the camera was adjusted manually and remained fixed for the duration of the experiment. Images were calibrated from the dimensions of the petri dish. Images were captured at a resolution of 5184 × 3456 pixels. Images were initially stored in the camera SD cards (Transcend) and subsequently transferred to a desktop computer hosting the image database, with images from each experimental batch stored in separate folders.

### Image database

**Fig. 3:**
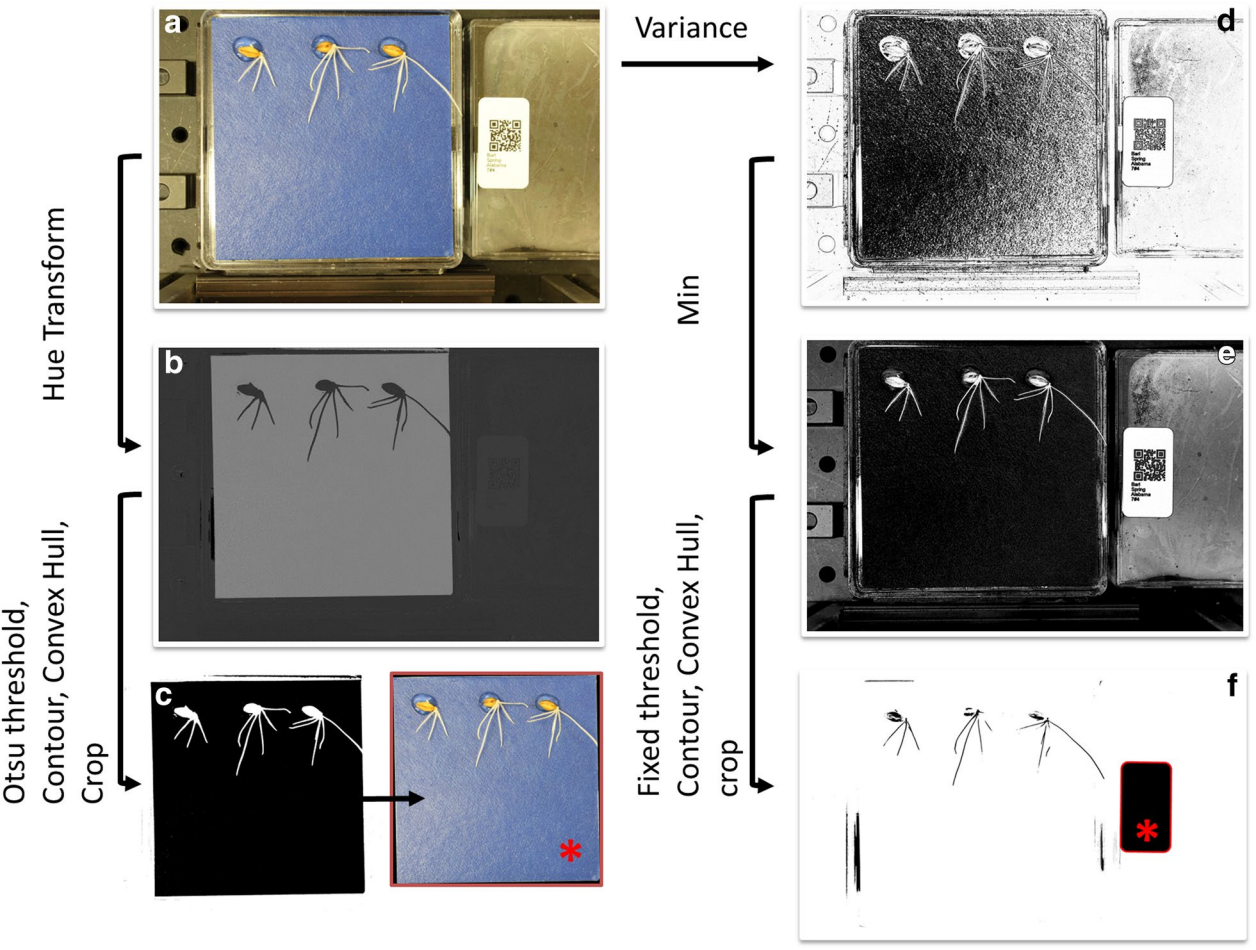
Two step image processing protocol for automated entry in the database. In the first step the raw images (**a**) are processed to identify the region of the blue germination paper containing roots (*column on the left*). This is achieved by determining the Hue value (**b**) of the image from the RGB data. The Hue image is then easily segmented using the Otsu auto-threshold approach and the contour of the region is then cropped (**c**). In the second step, the raw images are processed to identify and read the QR code in the image. This is achieved by determining the minimum image from the initial image data (**a**) and the image resulting from a variance filter (**d**). The resulting image (**e**) is then thresholded and cropped before it is passed into the Zbar decoder (**f**)

Raw image data were processed into a structured database and an image processing algorithm was used to identify the genotype corresponding to each image of the database (Fig. 3). The algorithm parses all the images and a series of steps are used to identify and read the QR code in the image: (1) images are first converted to 8 bit grey scale image; (2) a variance filter is then applied so that regions of the image characterised by a homogeneous distribution of pixel can be identified; (3) the minimum value between the variance image and the grayscale image is obtained so that only the regions with both homogeneous pixel distribution and with bright pixels remain with bright pixels; (4) the image is segmented using a fixed threshold of 80% of the maximum pixel intensity; (5) the sets of connected components in the thresholded images are then obtained using a contour algorithm; (6) the bounding box of the largest segmented component of the image (the QR code) is then used to crop the image and (7) the QR code is finally read and used to transfer the data to the appropriate folder. The information extracted at this stage is used to label images and folders in the structured dataset.

The region of the blue germination paper containing roots was identified using a second image processing algorithm: (1) the RGB colour image was first transformed into a Hue–Saturation–Value (HSV) colour image and the Hue value only was used to derive an 8 bit grey scale image; (2) the image was segmented with an Otsu auto threshold algorithm; (3) the contours of the largest connected component in the thresholded images was determined and (4) the exterior of the image was filled with black pixels and image cropped.

Finally, the raw image, the cropped image of the root system and the image of the QR code were copied into a set of hierarchical folders labelled with the corresponding species, population, genotype, and petri dish replicate number.

### Producing skeletons of root systems from the cropped images

Skeletons of root systems were obtained in a four step process. First, the location of seeds and root tips were marked on cropped images using customised software (http://archiroot.org.uk/). Two different markers were recorded in the image database, one for seeds and the other for root tips. Two methods for positioning markers were compared: One employed a conventional computer mouse to position the marks, whilst the other employed a manual stylet to position the marks using a 27 in. touch screen (1920 × 1080, Iiyama ProLite T2735MSC). Two batches of 28 image files were selected randomly from the image database. To avoid bias, on the first batch, markers were initially positioned using a conventional computer mouse and then using a touch screen computer and on the second batch, markers were initially positioned using a touch screen computer and then using a conventional computer mouse. A comparison of the times taken to position markers on an image using the two techniques demonstrated greater throughput of the phenotyping pipeline using a touch screen computer. 

Second, a suite of algorithms were employed to correct the position of any misplaced markers of seeds and root tips on cropped images. This procedure was followed:

1. The initial image (*I*) was transformed into an image (*J*) by multiplication with a kernel function centred on each of the markers. This operation produced a neighbourhood of candidate pixels that potentially improves the centring of the marker,
  1 $$J\left( x \right) = I\left( x \right)*\mathop \sum \limits_{1 \le i \le n} N\left( {x - x_{i} ,\sigma^{2} } \right)$$
  where x is vector of the coordinate of each pixel in the image, *N*(*µ*, *σ* ^*2*^) is the Gaussian function of mean *µ* and variance *σ* ^*2*^. *x*_*i*_ is the vector indicating the position of the centre of the *i*th marker.
2. Candidate positions for markers on the resulting image were obtained using an algorithm for the identification of local maxima (Michael Schmid, ImageJ). These operations were used to reduce the number of pixels to be considered for repositioning a marker.
3. Finally, the candidate position with the brightest intensity pixel was selected as the correct location for the repositioned marker.

The accuracy of positioning was defined as the distance between the initial placement of the marker and the placement of the re-positioned marker using the kernel centred algorithm.

Third, an optimal path algorithm was used to connect a seed to all root tips in the image. Various algorithms exists to perform such tasks, e.g. A* algorithm employed by RootNav [41]. Here, a classic Dijkstra’s algorithm was implemented [42]. The algorithm works by propagation of a front of pixels initiated at a seed. At each time increment, the growing front moves to immediately neighbouring pixels, and the cost of moving is recorded at each pixel. Because the entire image is explored, Dijkstra’s solution is not the most computationally efficient. However, it limits the chances of missing the global minimum of the error function.

The cost of a pixel is a function of pixel intensity expressed as a piece-wise linear function, and each pixel records the cost of travelling from the seed to the current position. The piece-wise linear cost-intensity function [43] used in this study is as follow:

2 $$E\left( I \right) = \left\{ {\begin{array}{*{20}l} {aI, } \hfill & \quad {I \le B} \hfill \\ {A\left( {I - B} \right) + aB,} \hfill & \quad {I > B} \hfill \\ \end{array} } \right.$$

The slopes of the piece-wise linear function *a* and *A* were chosen visually to minimize shortcuts in the path of minimal cost. *B* is the threshold at which the slope the cost function is increased. The pixel intensity *I* is such that roots are characterised with lower pixel intensities. The front stops at the limit of the images and the algorithm terminates once there are no pixels left in the front. The algorithm therefore produces a map of cost for an image. The map of the cost is then used to trace each root tip back to the seed. The path to the seed is obtained by descending the cost gradient until the seed is reached. This process is repeated for each seed in the image. At the end of this stage, each root tip is associated with three seeds.

**Fig. 4:**
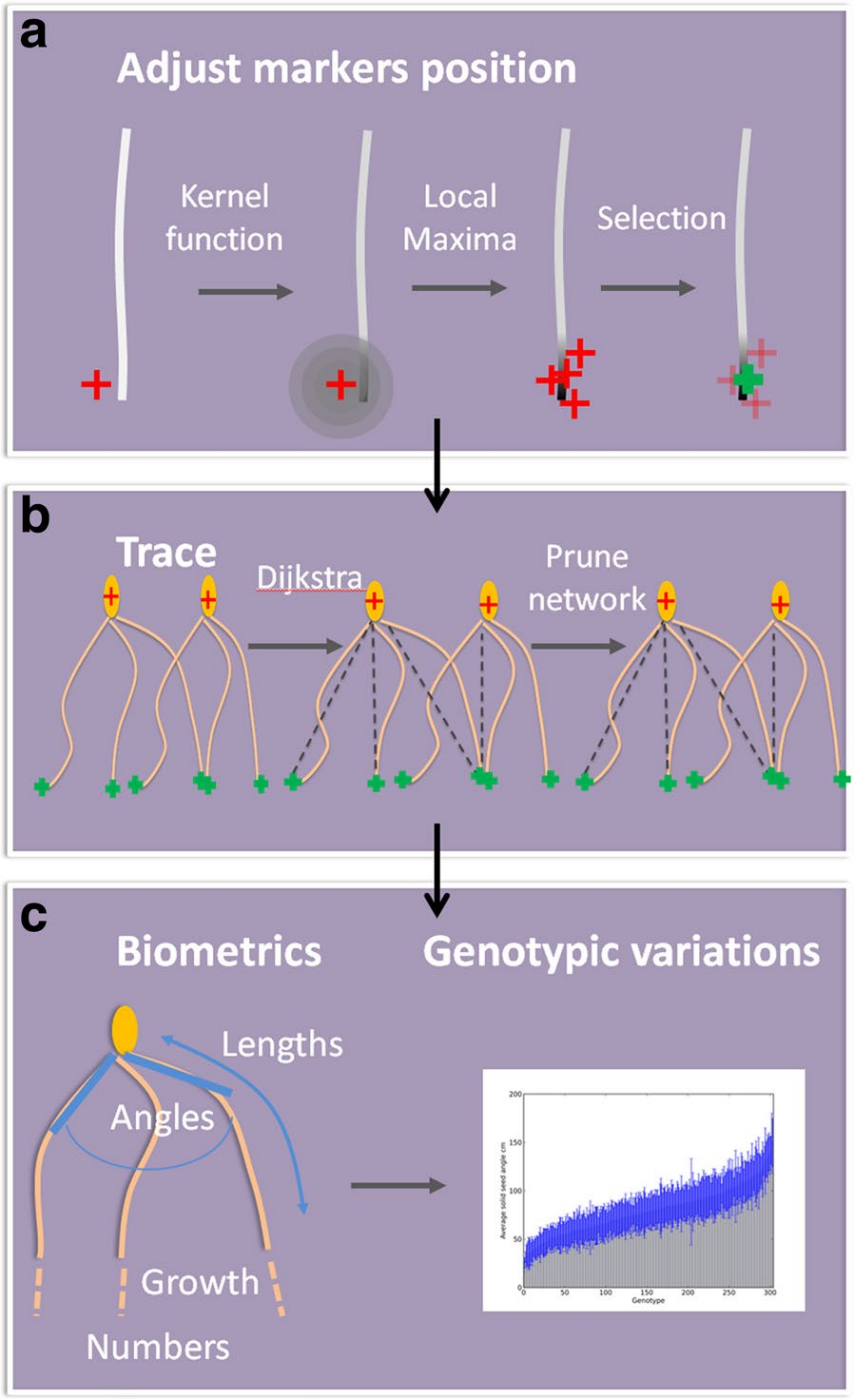
Extraction and analysis of rooting traits from image data. **a** The positions of the manually placed markers are corrected by a local search for optimal placement of the marker. The image is first transformed by multiplication of the image with a kernel function so the regions of high pixel intensity correspond to roots that are near a marker. Local maxima are then used to nearby candidates that are placed on a root. **b** The path of minimum cost between a root tip and a seed is established using the Dijkstra algorithm. Because there are several seeds in the image, the network of connections between seeds and tips is pruned so that only one seed is associated to a root tip in the image. **c** Root skeletons, which consist of a series of paths connecting seeds and tips, are analysed to determine root traits

Fourth, the total cost associated with each path between a root tip and a seed was determined. For each root tip, there were three possible paths connecting it to the seeds in the image (Fig. 4). The path with minimum cost was used to associate a root tip with its corresponding seed.

For each image, three root system skeletons were obtained. Each skeleton comprised a set of paths. Each path represents a root that connects a seed to a root tip and is described as a list of coordinates of connected pixels. The coordinates of the root system skeleton were recorded in an output file associated to each cropped image in the image database.

### Determining root architectural parameters from skeletons of root systems

The list of coordinates representing the root skeleton was used to determine a variety of root architectural parameters. For barley, the number of germinated seeds, number of roots per seed, length of individual roots, length of the longest root, total length of the root system, angle of spread of roots at the seed and angle of spread of the root system at the root tips were calculated.

The number of germinated seeds was determined as the number of seeds associated with at least one root tip. The number of roots per seed was determined as the number of tips associated to a seed. The length of individual roots was calculated as the sum of the Euclidean distances between consecutive pixels in a path. The length of the longest root was calculated as the sum of distances between consecutive pixels in the longest path from an individual seed. The total length of a root system was calculated as the sum of the lengths of individual roots from a particular seed. The angle of spread of roots at the seed (basal solid angle) was calculated as the difference between the angle of the outermost root on the right and the angle of the outermost root on the left. The angle was calculated based on segments connecting the seed and the point placed at a distance of 10% of the length of the total root. The angle of spread of the root system at the root tips (apical solid angle) was calculated as the difference between the angle of the outermost root on the right and the angle of outermost root on the left. The angle was calculated based on segments connecting the seed and the tips of these two roots.

For oilseed rape, the number of germinated seeds, number of roots per seed, length of the primary root, angle of the primary root from the vertical at the seed and angle of the primary root from the vertical at the root tip were calculated. The number of germinated seeds was calculated as the number of seeds associated with at least one root tip. The number of roots per seed was determined as the number of tips associated with a seed. The length of the primary root was calculated as the sum of distances between consecutive pixels in the longest path. The angle of the primary root from the vertical at the seed was calculated from the segment connecting the seed and the point placed at a distance of 10% of the length of the primary root. The angle of the root from the vertical at the root tip was calculated based on the segment connecting the seed and the tip of the primary root.

Summary files containing the root architectural parameters for each seedling were produced from this analysis. These were stored as csv files in the top level folder.

### Analysis of variations in root architectural parameters

A complete set of root traits (number of roots per seed, total root length, average root length, length of the longest root, basal solid angle, apical solid angle, number of germinated seeds) was obtained for all the images of the root database and for each root system. Thus, the maximum number of replicates for a genotype of which all seeds germinated was 15. For each root trait the mean and standard error of the mean (SE, n ≤ 15) were calculated for each genotype.

### Estimates of throughput of the phenotyping pipeline

**Fig. 5:**
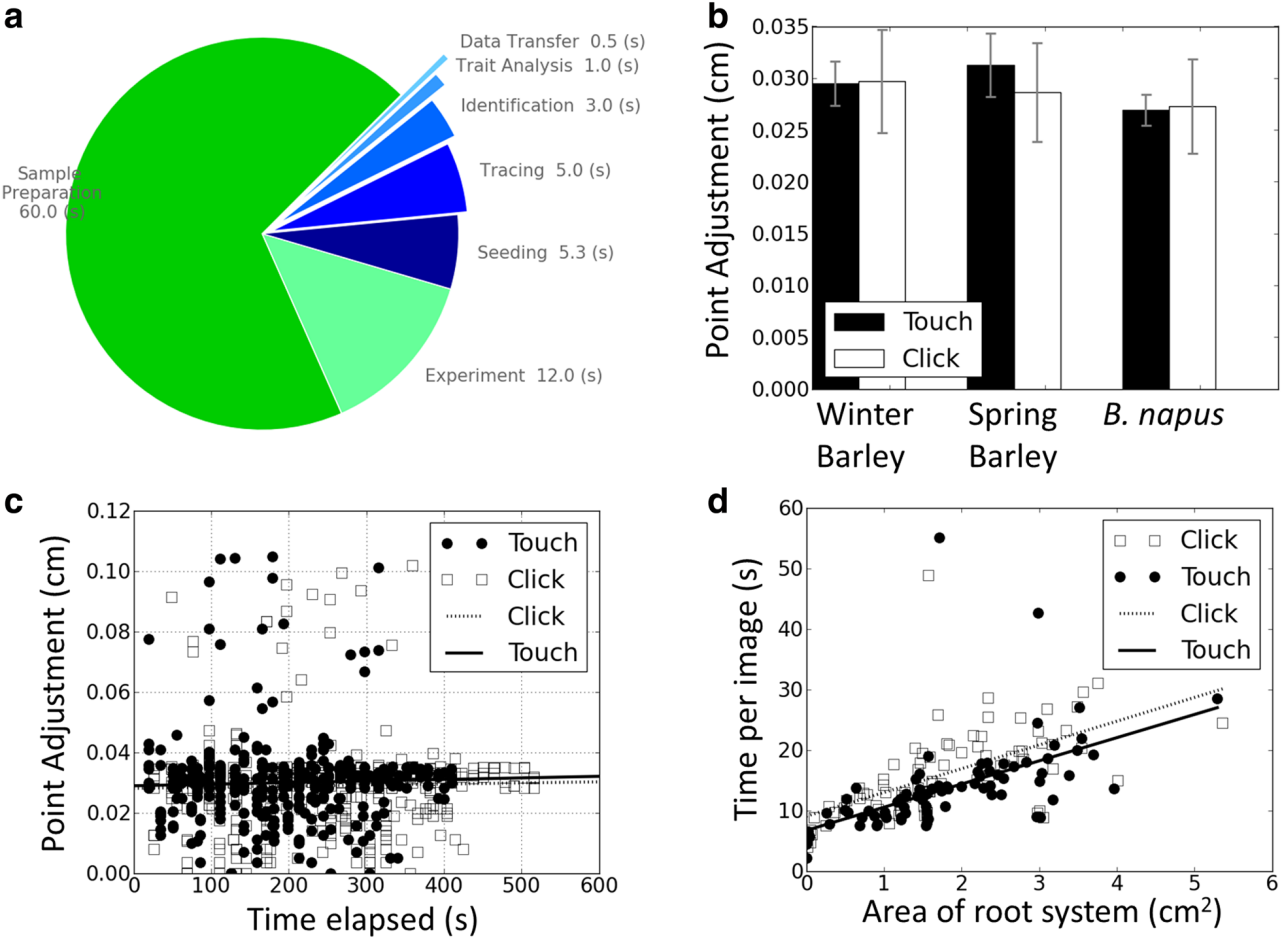
Time and error analysis of the phenotyping pipeline. **a** Times taken to process one seedling divided into the seven steps of the root phenotyping process. In decreasing order of time, the steps consist of (1) sample preparation, (1) experiment (manual operations required to move samples, capture image data and watering), seeding (manual placement of markers on images), tracing of roots, identification and reading of QR codes, Analysis of root traits, and transferring files into the database. **b** Adjustments of markers as a function of method of marker placement and species. **c** Adjustments of markers as a function of time. **d** Time required for marker placement as a function of method of marker placement and area covered by the root system

The phenotyping pipeline was divided into seven steps: (1) preparing petri dishes, (2) handling samples during imaging, comprising both image acquisition and watering, (3) transferring raw images to the database, (4) reading barcodes and populating hierarchical folders, (5) placing markers on images, (6) producing skeletons of root system architectures and (7) extracting trait data from skeletonised root architectures. The throughput of each step of the phenotyping pipeline was expressed as the number of seeds or seedlings processed per unit time. This was estimated from the time of processing a specific number of samples. The times taken to process one seed or seedling in each step were compared to identify bottlenecks in the phenotyping pipeline (Fig. 5). The time required for the analysis of the image data was compared with that required to trace root data using the RootNav software [41]. The time required to place the markers, the time required to correct the errors of the tracing, and the time of image processing (including opening files and operating the user interface) were recorded for the RootNav software.

### Estimating types, frequency and effects of errors on skeletons of root systems

Several types of errors were observed in the automatic skeletonisation of root systems. These included:

(Error 1) Path not on a root (“shortcut”). This can be caused by (a) misplacement of a marker or (b) roots of high curvature. This is an inherent limitation of Dijkstra’s algorithm. The effect of the latter error is usually to underestimate the length of the root being traced. The shortcuts usually occur over a small distance where roots bend and does not induce large changes in root length.

(Error 2) Two paths converge on a single root (“convergence”). This occurs when two roots cross or when the path of two roots converge and the minimal cost is obtained for only one of the paths. When this error occurs, the length of the root is usually underestimated although in some cases longer brighter roots can be prone to multiple tracing. This could be corrected by improved algorithm introducing cost for “sharing” a path.

(Error 3) A root tip is associated with the wrong seed (“wrong association”). This occurs when the root tip of a seedling is very close to the tip of another seed or when the marker for a root tip is misplaced. These errors can induce large inaccuracies in the measured root length. Although our software does not do this, these errors might be detected easily since they induce changes in the number of roots per seed and can be corrected simply by imposing the correct association between the tip and the seed.

(Error 4) Placement of a marker for a root tip away from the root tip. There are three types of “misadjustment”: the marker is on the wrong root (“misplacement”), the marker is not on a root (“not on root”), or the marker is on the right root but not exactly at the tip (“not on tip”). Markers for root tips are usually misplaced by a small distance. This error in itself does not constitute a major problem. However, misplacement of marker for a root tip can be the source of other errors (e.g. Errors 1–3). Misplacement of markers can be corrected by manual adjustments once the error is detected.

The frequency of these errors was examined on 90 images selected randomly from each mapping population contained in the database (30 images of Winter Barley, 30 images of Spring Barley and 30 images of oilseed rape). For each of these images, the number and types of error were recorded. This data was used to determine the fraction and frequency of each type of error. The severity of these errors was also estimated globally using 30 images selected randomly from each mapping population. For each of these images, total root length was determined using four alternative algorithms: the tracing algorithm from SmartRoot software [15], Fiji’s Simple Neurite Tracer based on a similar live wire algorithm [44], RootNav [41], and using maximum entropy segmentation followed by skeletonisation [45]. For RootNav analyses and maximum entropy segmentation, images were pre-processed prior to analysis. First, only the red channel of the images was used for image processing. Short range variations were corrected using a median and Gaussian filter of radius 0.16 mm to produce an initial image. Images were then corrected for long range variation (background subtraction) by obtaining a background image using a median filter of diameter 2.6 mm and subtracting the value of the background image from the initial image. For RootNav analyses, images were scaled down to 1:2 prior to image processing to improve processing efficiency. A correlation matrix was then obtained to examine similarities of the results provided by the different methods.

### Software and statistical analysis

Image analysis tools were implemented as two distinct ImageJ macros for the identification of the growth zone and the QR code. An ImageJ plugin was also written to adjust the position of markers and produce the skeletons of root systems from the cropped images. Decoding of bar codes was performed using the Zbar library 0.10 (www.zbar.sourceforge.net). These different components were assembled together in a customised software package named ArchiPhen. The ArchiPhen software decodes the QR codes in the image, crops the growth zone and imports data from experiments into a hierarchical folder structure based on species, population, genotype, replicate number and time. Once in the database, ArchiPhen was also used to select seed and root tip markers on the image, run image analysis plugins for tracing the skeletons of all root systems in the database, visualize skeletons, and correct potential errors. The interface was constructed using the Python 2.7 programming language (www.python.org). The source code is freely available at www.archiroot.org.uk. Statistical analyses were performed using the R software package [46]. Two-way ANOVA was applied to study the effect of species and method of placement of marker (click or touch) on the errors of placement (Fig. 5). Analysis of covariance was applied to study the effect method of placement of markers (click and touch) on the time to perform the task, and to study the effect of habituation on the errors of placement of the markers. Analysis of covariance was also applied to compare the accuracy of the different software for root tracing.

## Results and discussion

The purpose of this work was twofold: (1) to describe a cost-effective root phenotyping pipeline, incorporating low-cost infrastructure and custom software, and (2) to perform time and accuracy benchmarking of this phenotyping pipeline to identify bottlenecks in such pipelines and strategies for their acceleration.

### Low cost HTP system: a time-and-motion study

A low-cost, computer-assisted, root phenotyping pipeline was established (Fig. 1), which is similar to that described by Goioa et al. [21]. The infrastructure required is modest and common to most laboratory environments. It comprises a temperature-controlled incubator, a camera with a custom-made stand, and a computer (Fig. 2). Algorithms are then used to locate and read the QR code and analyse the morphology of the root from image data (Figs. 3, 4). The costs of running the pipeline are also modest, comprising petri dishes, filter paper and some basic chemical reagents (Fig. 2). At present these cost about £0.8 per plant (1.04 USD on 16 June 2017). Staff time approximates 1 min person per plant preparation time, 12 s person per plant handling time during the experiment, and <15 s person per plant for all image processing (Fig. 5a). The image processing is facilitated by customised computer software (ArchiPhen; http://archiroot.org.uk/). This rapid pipeline has a theoretical throughput of about 320 plants per person per 8 h working day (sum of all time components/8 h). This allowed 12 replicates of 204 oilseed rape (in 5 batches), 304 Spring barley (in 8 batches) and 282 Winter barley genotypes (in 5 batches) to be phenotyped in a 6 month period for the studies reported here. The preparation of petri dishes and handling of plants are by far the most time-consuming processes. At present staff tasks cannot easily be replaced by robotics. However, greater speed and accuracy in image processing can be achieved using customised computer software.

A detailed time-and-motion analysis of the image processing steps of the pipeline indicated that (1) there was no effect in the accuracy of the placement of markers on root tips caused by the species analysed (p = 0.27) or, the method of marker placement (touch screen or computer mouse; p = 0.39) nor any interactions between these (Fig. 5b; p = 0.45), (2) there was no change in accuracy with time over a 45 min period (p = 0.42) in the placement of markers on root tips, and this was not affected by the use of a touch screen or a computer mouse (Fig. 5c, p = 0.31), (3) using a touch-screen, rather than a computer mouse, significantly accelerated the placement of markers by 10% (Fig. 5d; p = 0.006), and (4) the main factor affecting the time to place markers was the area occupied by the root system on the image (Fig. 5d; p < 0.001). The placement of markers was the most time consuming step of the image analysis (Fig. 5a). Since manual positioning using a stylet and touch screen was the most rapid and ergonomic, this method was subsequently used to process all cropped images. In the future, the placement of markers on an image might be performed automatically using algorithms that locate root tips and seeds automatically [47].

Analyses were also carried out to compare the time required to segment images using ArchiPhen with that required using RootNav [41]. RootNav is a software tool that uses techniques similar to ArchiPhen for tracing roots but also the capability for manual error correction. Results showed that the time required to place markers was similar for both software tools: 4.7 ± 0.38 s per plant were required to place markers using RootNav (mean ± SE, n = 20) while 5.3 ± 0.02 s per plant were required to place markers using ArchiPhen (mean ± SE, n = 84). However, the time for image processing was 23.8 ± 2.03 s per plant using RootNav (mean ± SE, n = 20) whereas it was only of 5.0 s per plant using ArchiPhen. The time required to correct the errors of the tracing was 30.7 ± 5.95 s per plant using RootNav (mean ± SE, n = 20). Although the time to correct errors for ArchiPhen was not recorded, both software tools produced similar types and frequencies of errors. Therefore, it is reasonable to expect that, using the same graphical user interface, a similar time would be required to correct all errors after running ArchiPhen.

### Analysis of software errors

**Fig. 6:**
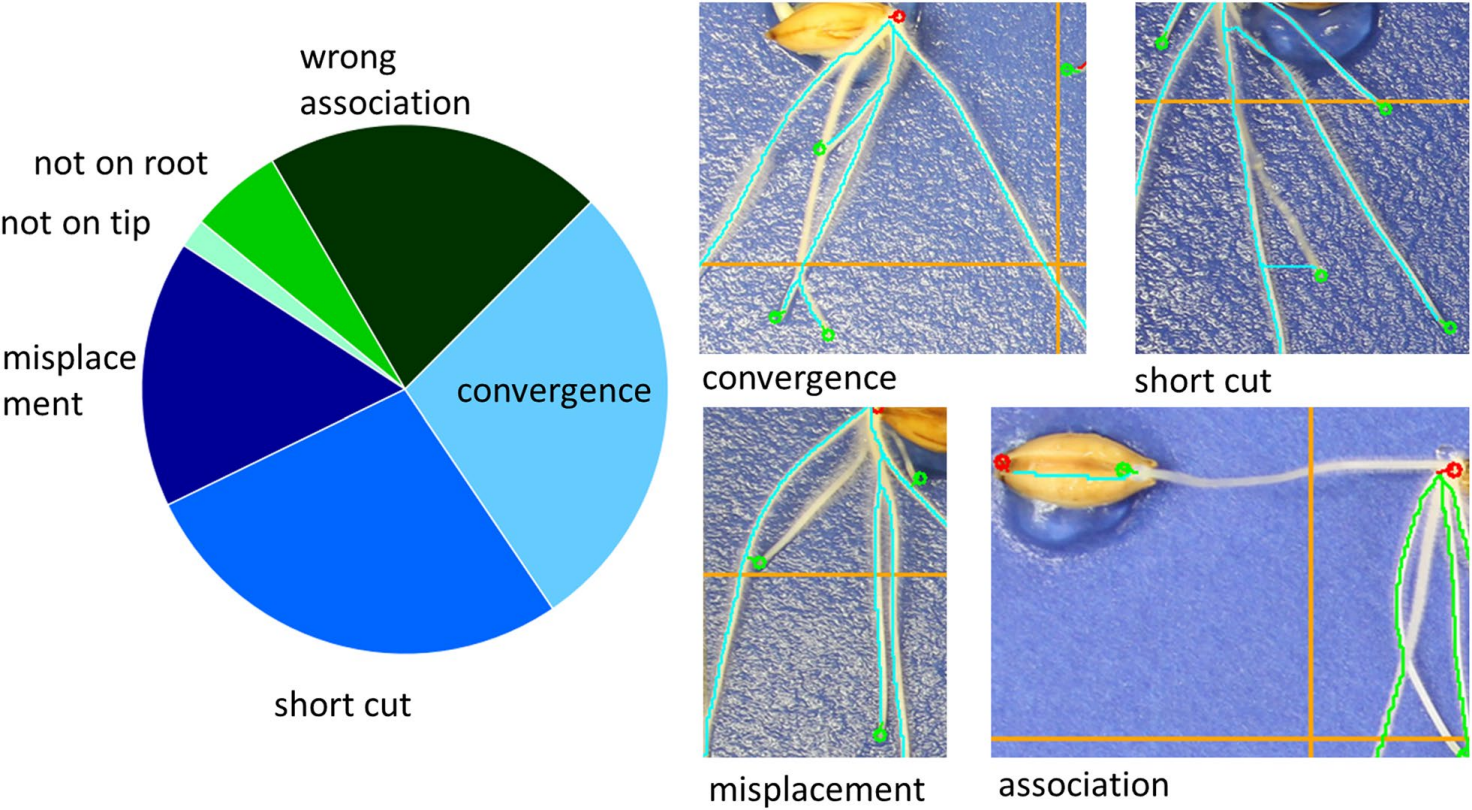
Root tracing errors in the database. The analysis of the frequency of errors shows the most common types of errors are due to “short cut” and “convergence” errors, which allow for multiple possible root tracings. Other errors are due to “wrong associations” between seed and root tip or the “misadjustment” of a marker for a root tip causing (1) large errors when a neighbouring root is traced (“misplacement”), or (2) small errors when the correct root is traced but the marker is placed either on a root, but not on the root tip, or not on a root

Several errors were observed in image analysis (Fig. 6). These included (1) mistracing of roots resulting from a “shortcut”, which occurs either because the root is curved or lacks contrast with the background, (2) mistracing of roots due to them “converging” with each other, which allows for multiple possible tracings, (3) “wrong association” of a root tip with a seed, which often occurs when a root tip or its marker is in contact with a neighbouring plant and (4) “misadjustment” of a marker for a root tip causing either large errors when a neighbouring root is traced (“misplacement”), or small errors when the correct root is traced but the marker is placed either on a root, but not on the root tip, or not on a root (Fig. 6). In general, the number of images generating errors in image analysis was greater when more complex root systems were analysed. About 60% of the images of barley root systems generated errors in image analysis, whereas only 15% of the images of brassica root systems generated errors in image analysis. The frequencies of the compared four types of errors were similar (Fig. 6). Hypothetically, each type of error can be overcome to some extent either by additional human intervention or improvements in the computer software.

**Fig. 7:**
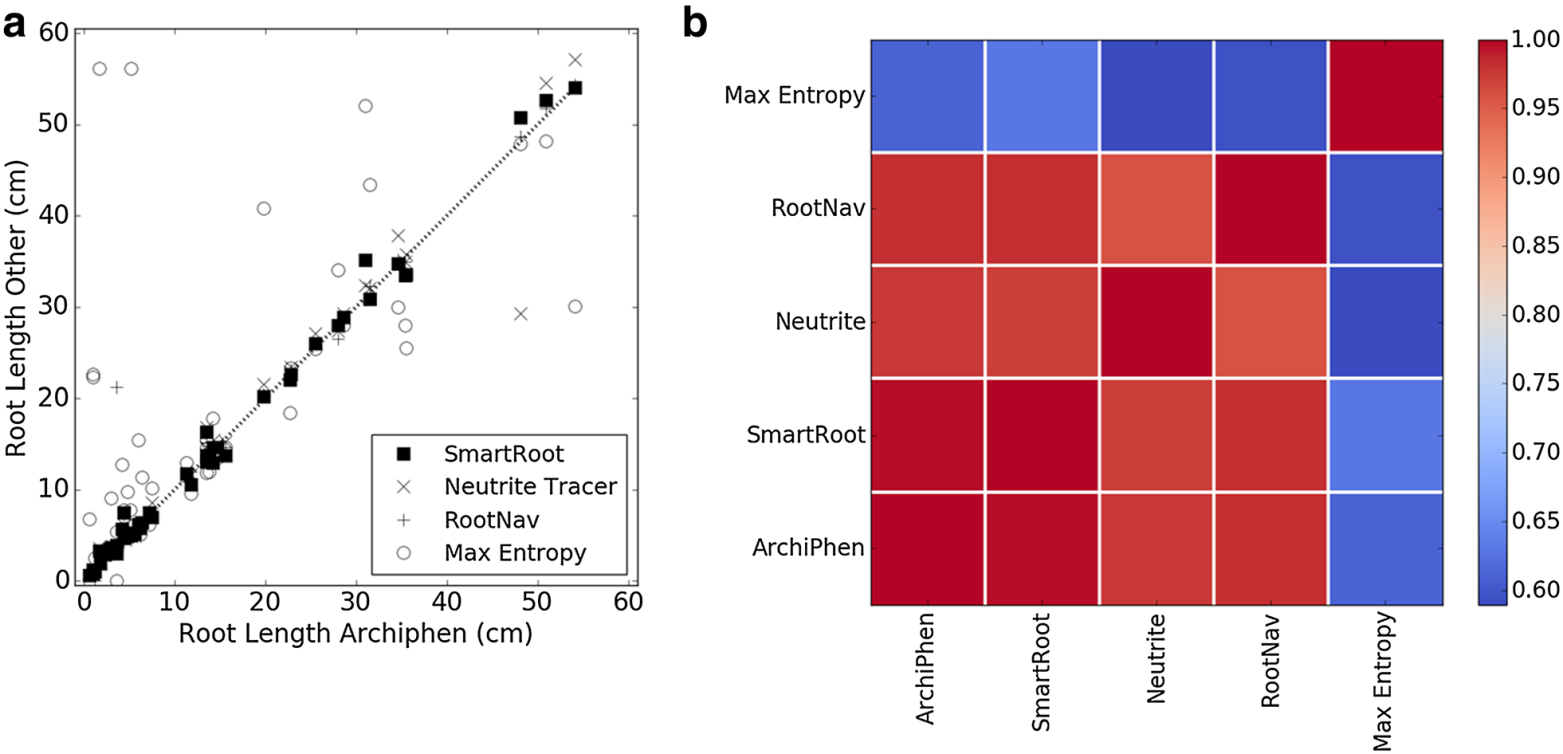
Benchmarking results from image analysis **a** comparing root lengths measured by ArchiPhen with those of commonly used software, SmartRoot (*squares*), Simple Neurite Tracer (*crosses*) and a simple Maximum Entropy auto thresholding method (*circles*) and **b** a correlation matrix of similarities of the results provided by the different methods

The mistracing of roots of high curvature as a shortcut is a consequence of path optimality being defined in terms of the total cost of a path. Thus, the algorithm generally favours paths that are shorter [48]. Such errors are usually corrected by forcing a path through defined points in regions of high curvatures [44, 49], but this can increase the time spent by an operator on a single image significantly. The effect of this error is to underestimate the length of the root being traced. However, since shortcuts usually occur only across small distances, this does not create large misestimates of root length (Fig. 7).

The mistracing of roots when they cross each other occurs because the subsequent tracing proceeds along a common path of minimal cost. This error often results in an underestimate of root length, although longer, brighter roots can be prone to multiple tracing. In the past, various modifications of the optimality criteria have been introduced to correct for mistracing. In particular, various methods of weighting the cost of a path have been used, for example using various internal cost functions combining the Eigen values of Hessian matrix as an indicator of the direction of the object [50] or gradient, Canny, and or Laplacian of Gaussian of the image [49, 51]. Training the error function “on the fly” has also been successful to tailor the error function to the nature of the object to be traced [52]. In this case however a different approach is required because error function accounts only for single tracing. Such errors might be corrected either by human intervention in forcing alternative paths or by introducing a cost for sharing the same path. Alternatively, a single petri dish may be used for a unique seedling or more images might be taken at a greater frequency, although these would increase both the cost and time of manual operations.

The misassociation of a root tip with a neighbouring seed can create large errors in estimates of root length. However, these errors can be detected easily in histograms showing the number of roots per seed and can be corrected simply by stipulating the correct association between a root tip and the seed. Misadjustment of markers for a root tip is usually only by a small distance, but can have large effects on estimates of root length. These errors can be corrected either by human adjustment of the marker for the root tip once an error is detected or using computer software, for example using corner detection algorithms [53, 54].

Many approaches to computer-assisted root tracing have been published recently. Segmentation by thresholding and skeletonization has been employed in software such as EasyRhizo and GIA root [55, 56] or WinRhizo (Regent Instruments Inc., Ottawa, ON Canada). Tracking has been employed in SmartRoot [15] and in the TubularTracking plugin of the Mevislab software [57] used by Downie et al. [12]. Optimal path searching has been used in RootNav [41], Simple Neurite Tracer [44] and in ArchiPhen (this study). These three approaches for extracting root architectures from image data have been compared (Fig. 7a) as (1) the fully automated Maximum Entropy auto-thresholding method [58], (2) the SmartRoot automated individual root tracing algorithm based on a tubular tracking approach [15], (3) the Simple Neurite Tracer, which is based on a path searching algorithm [44] and (4) RootNav [41]. Maximum Entropy was the fastest, but the least accurate, method. It suffered from both false detection of roots and inability to detect true roots. The SmartRoot, Simple Neurite Tracer and ArchiPhen methods all gave similar results (Fig. 7b). The correlation coefficient (R^2^) between ArchiPhen and SmartRoot was 0.99 (p < 0.001), between ArchiPhen and Simple Neurite Tracer was 0.98 (p < 0.001) and between ArchiPhen and RootNav was 0.96 (Fig. 7, p < 0.001). Correlation between ArchiPhen and Maximum Entropy thresholding was 0.68 (p < 0.001).

### Streamlining root phenotyping pipelines

It is evident that root phenotyping has been accelerated through the development of computer software for tracing roots and by comparing their performance [47, 56, 59]. However, further improvements to the accuracy of tracing, and the speed at which tracing is performed, are likely to be of limited benefit. Most recent methods provide similar accuracy (Fig. 7), and the processing time for tracing roots is short compared to other tasks required for phenotyping root systems, particularly the manual tasks (Fig. 5). To increase the throughput of root phenotyping pipelines, focus might be redirected towards minimising human interventions. Current software relies on a human operator performing various tasks, for example: opening image files, manual identification of regions of interest within the image, iterative corrections of errors in outputs until a suitable representation of the root system is obtained. Optimising these interactions could increase throughput and facilitate the screening of large plant populations. In addition, greater automation throughout the phenotyping pipeline could minimise human interventions elsewhere and reduce the burden of repetitive tasks.

There has been little research to quantify and optimise human interventions in phenotyping pipelines. Here, a detailed time apportionment study was conducted using a stereotypical pipeline for phenotyping plant roots (Fig. 5a). This study indicated that sample preparation and plant husbandry were the most time consuming tasks, but that a significant amount of time was spent interacting with computer software. To minimise these interactions attempts were made to reduce time spent by staff on correcting errors during image analysis and to perform computations in large batches without the necessity for human intervention. Thus, the pipeline for image analysis was adapted (1) to facilitate the transfer of image data into a structured database by automating the reading of barcodes and location of the root system in raw images, (2) to minimise the time required by staff to place markers on images using touch screen technology, (3) to prevent staff spending time correcting mistakes in image analysis during the placement of markers and (4) to perform computational analyses for all images in a single batch. In addition to these innovations, the pipeline might be accelerated by taking pictures of several boxes simultaneously, although this would require more skilful manual intervention, and storing images immediately on computer rather than transferring them from a camera SD card. Future work should focus on identifying and correcting tracing errors a posteriori, and, when possible, do this without human intervention.

Further acceleration of this root phenotyping pipeline might lie in the ability to correct tracing errors automatically in an unsupervised manner. Some errors might be identified automatically by searching for plants with apparently abnormal morphological characteristics. For example, (1) histograms of root numbers per seed could be used to identify plants in which root tips are associated with the wrong seed, (2) histograms of root length per plant could be used to identify large mistracing errors, and (3) histograms of root tortuosity per plant could be used to identify “crossing” and “short-cut” errors. However, since extreme phenotypes might confound the analysis of individual histograms, and each histogram provides different information, the most reliable method to identify errors would require a combined analysis of multiple types of histograms. This strategy would identify errors that have large effects on estimates of root traits. However, most errors observed in this study had little consequence for estimates of root length per plant (Fig. 7), root number per plant, or the angle of root growth (data not shown). The identification of errors with small effects on trait estimates might require more sophisticated strategies. Techniques based on machine learning have proved extremely successful at automatic identification and classification of patterns [60, 61]. The simple typology of errors established here (Fig. 6) will facilitate the application of such techniques.

In summary, we have described (1) a low-cost HTP system for phenotyping seedling root architecture, (2) a semi-automated image capture and curation pipeline, (3) a new algorithm for correcting manual placement of markers on seeds and root tips and an shortest path algorithm similar to other tracing software, such as RootNav [41], for tracing roots without prior knowledge of relationships between seeds and root tips, that allow data on root number per seed, root length per seed and root angles to be estimated rapidly for large populations with minimal user input. Time-and-motion studies indicate that sample preparation and handling of samples during screening are the most time consuming tasks in the root phenotyping pipeline. Future engineering efforts might seek to automate these tasks. Benchmarking the image analysis software developed for the pipeline, ArchiPhen, with other image analysis software suggested that it performed comparably. Although software can be used to speed up the extraction of root traits from image data, when applied to large numbers of images there is a trade-off between the time of processing data and errors contained in the database. The next step would be to implement error detection systems and accelerate the image analysis by developing faster and more reliable replacement for manual interventions.
